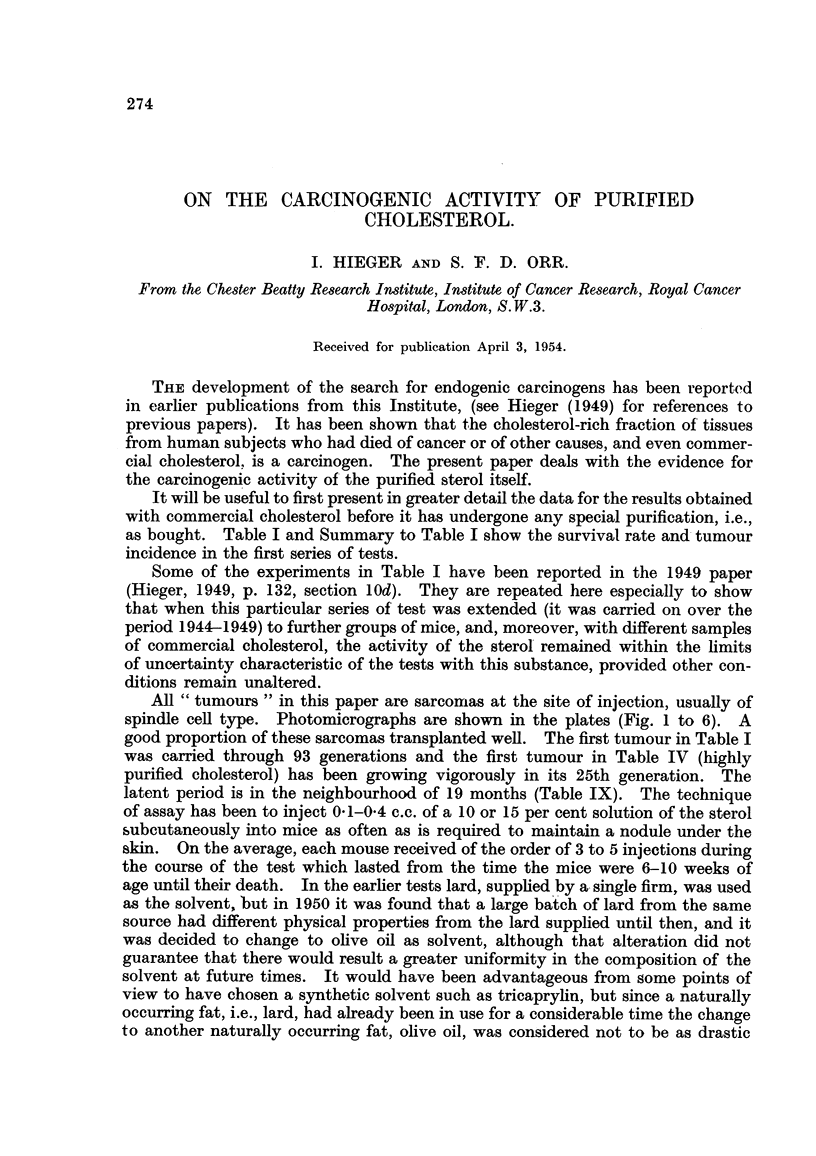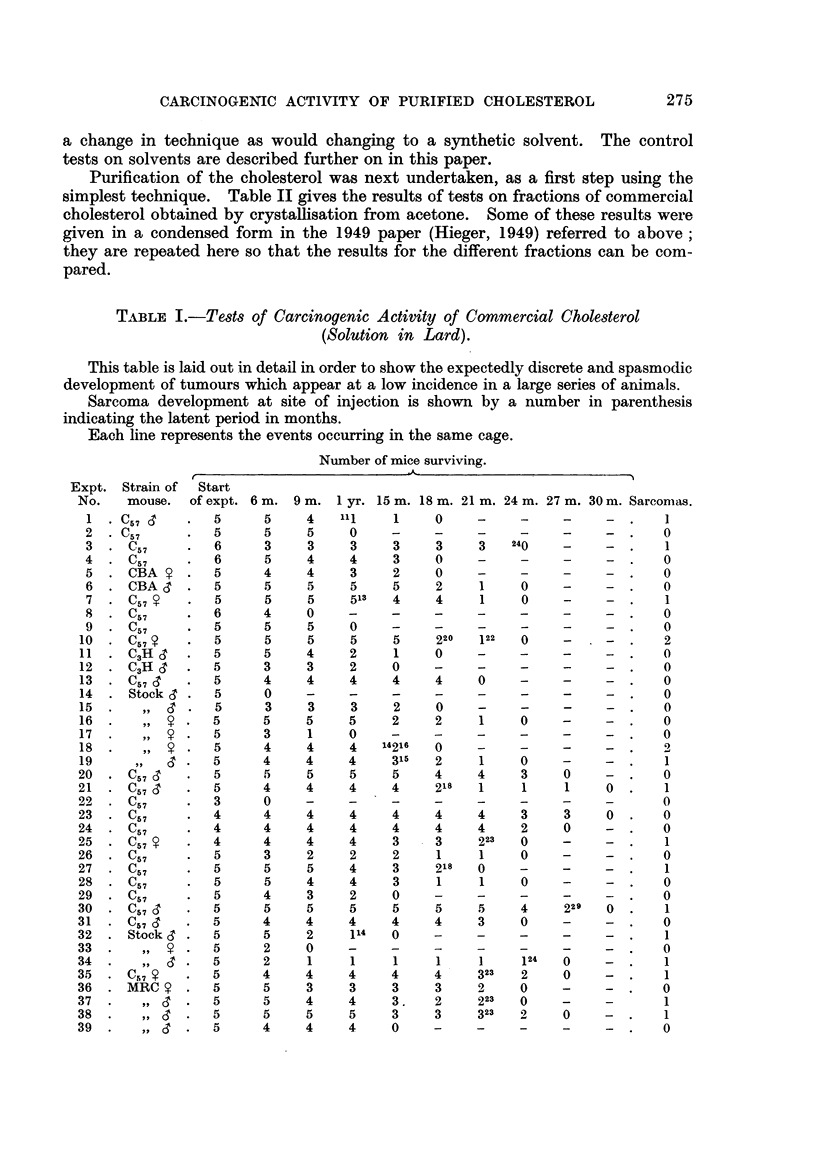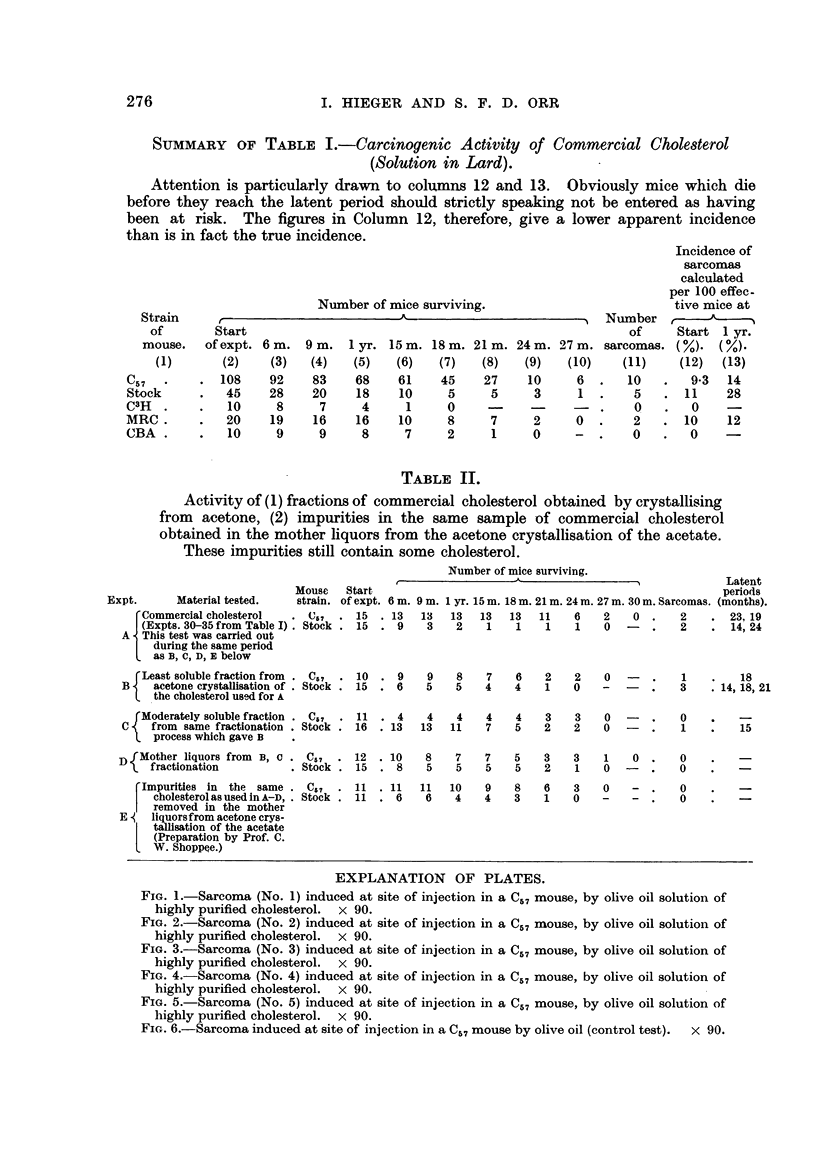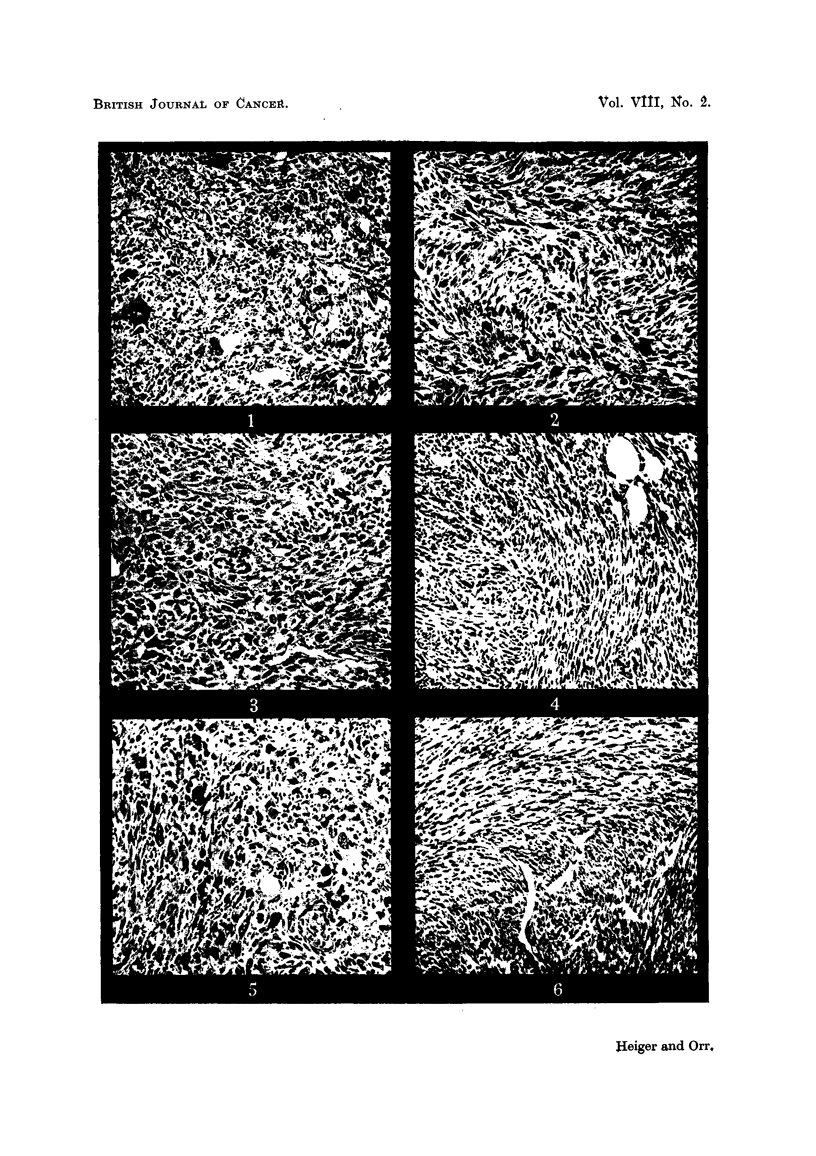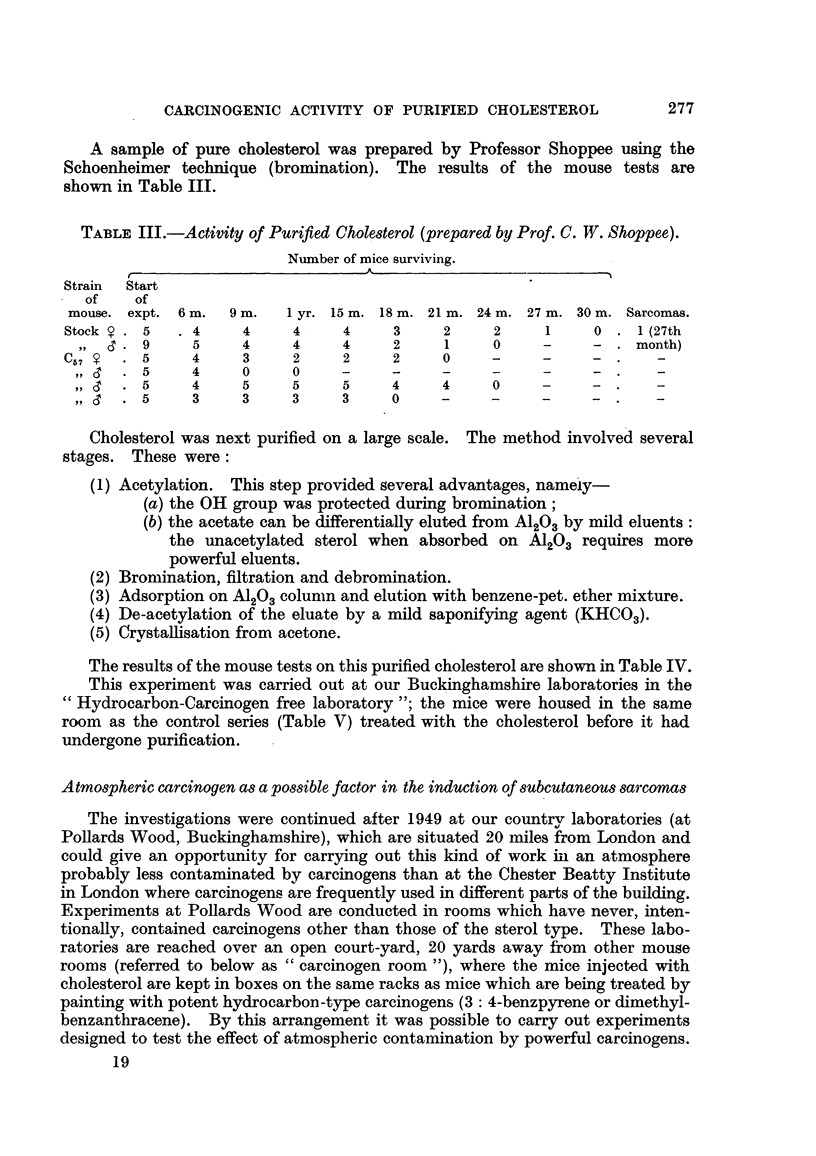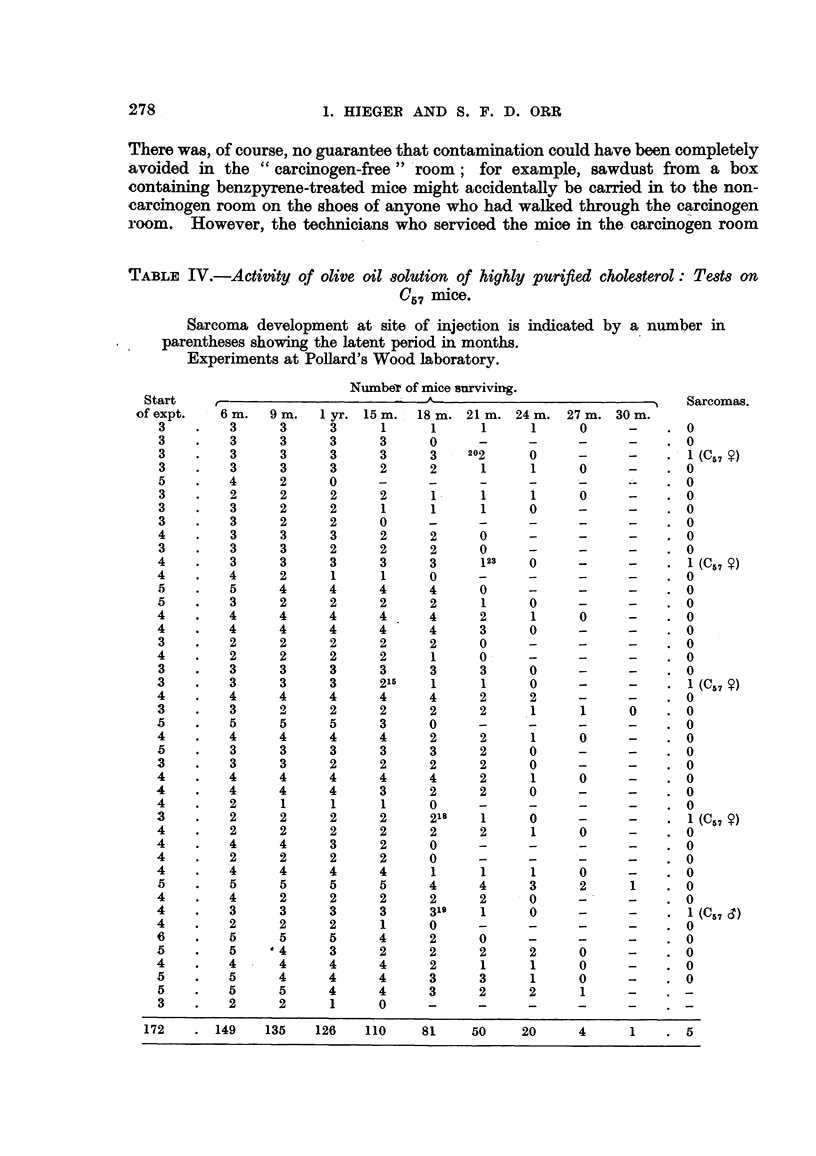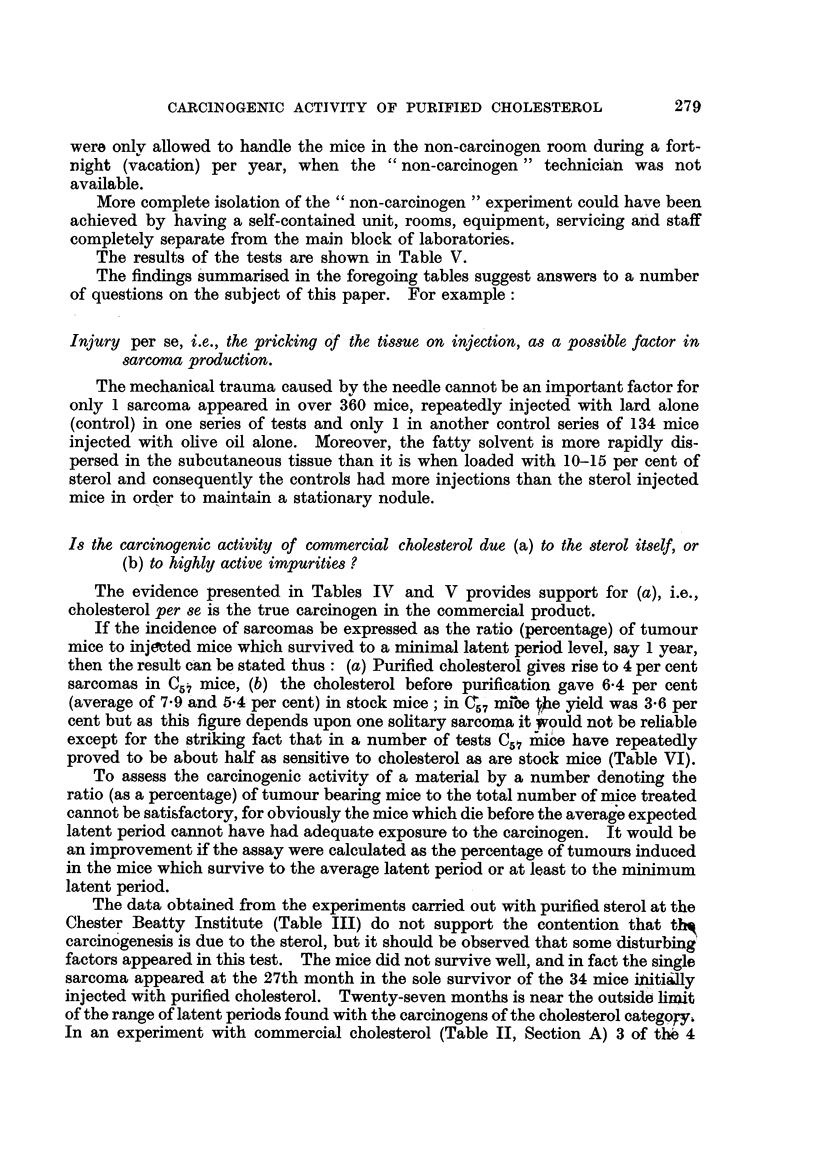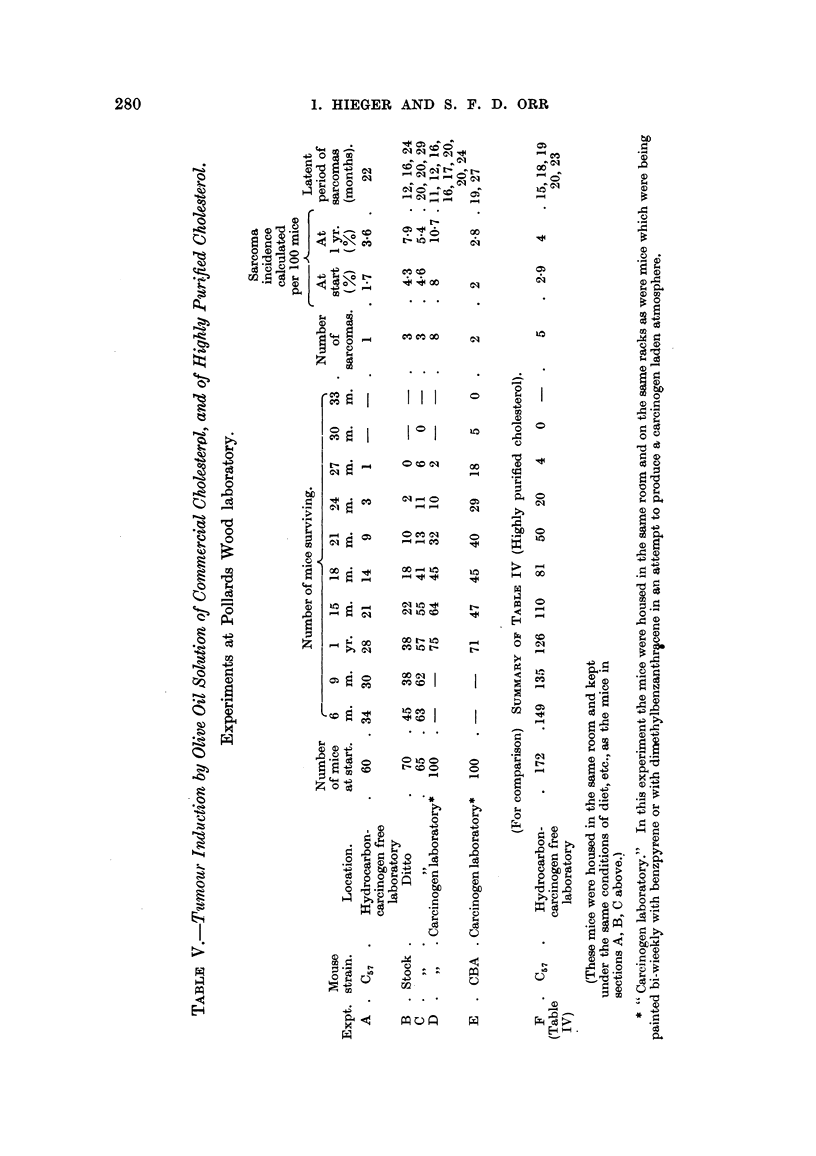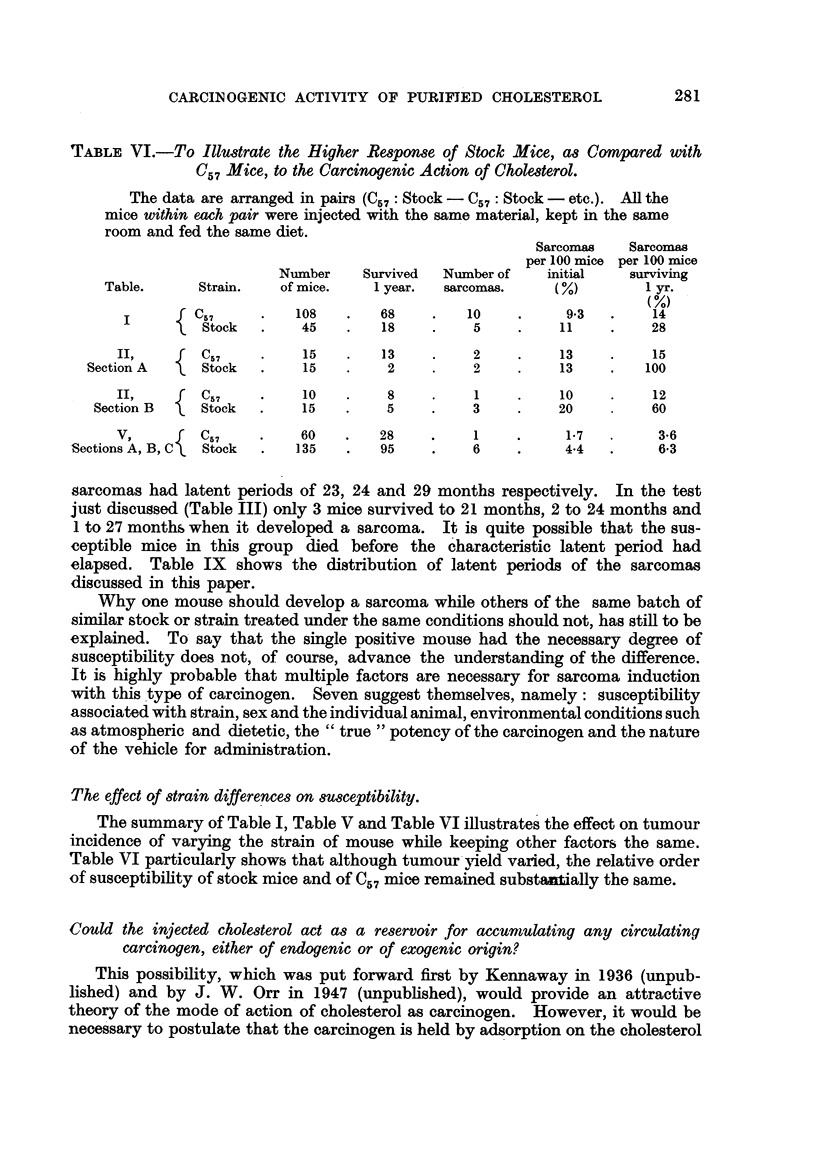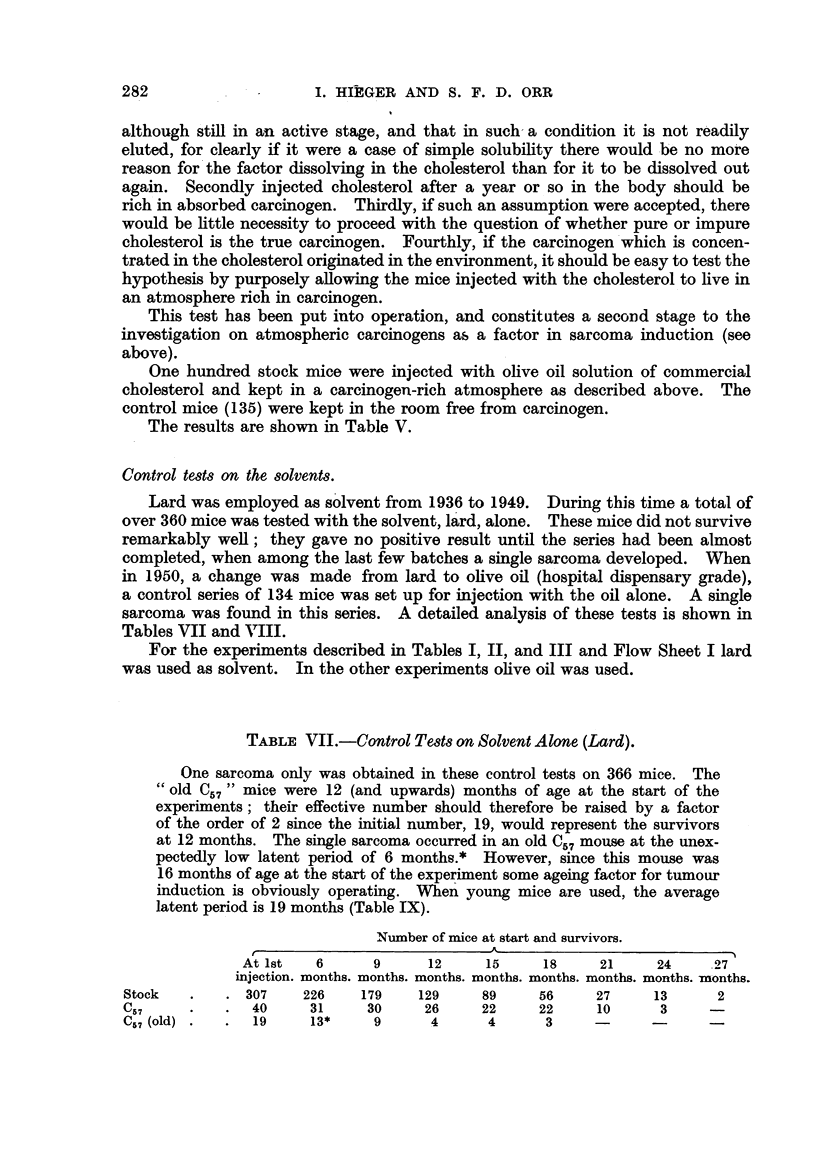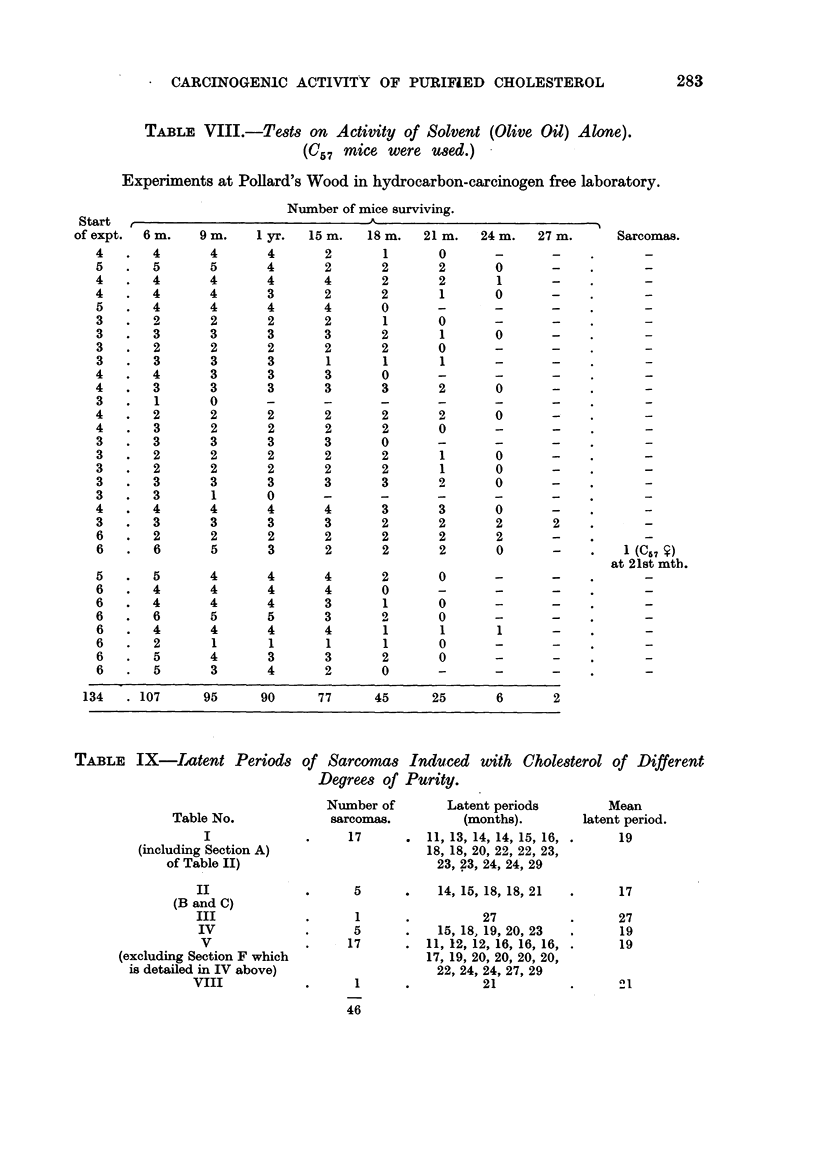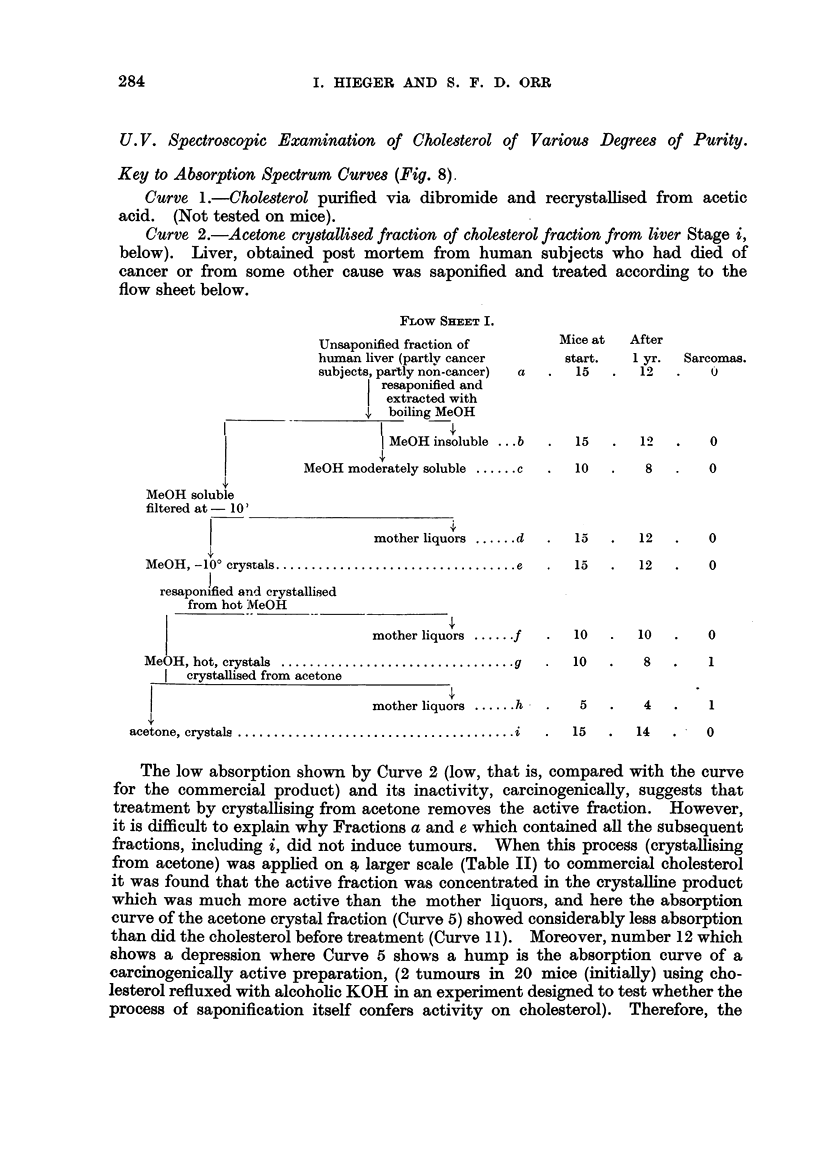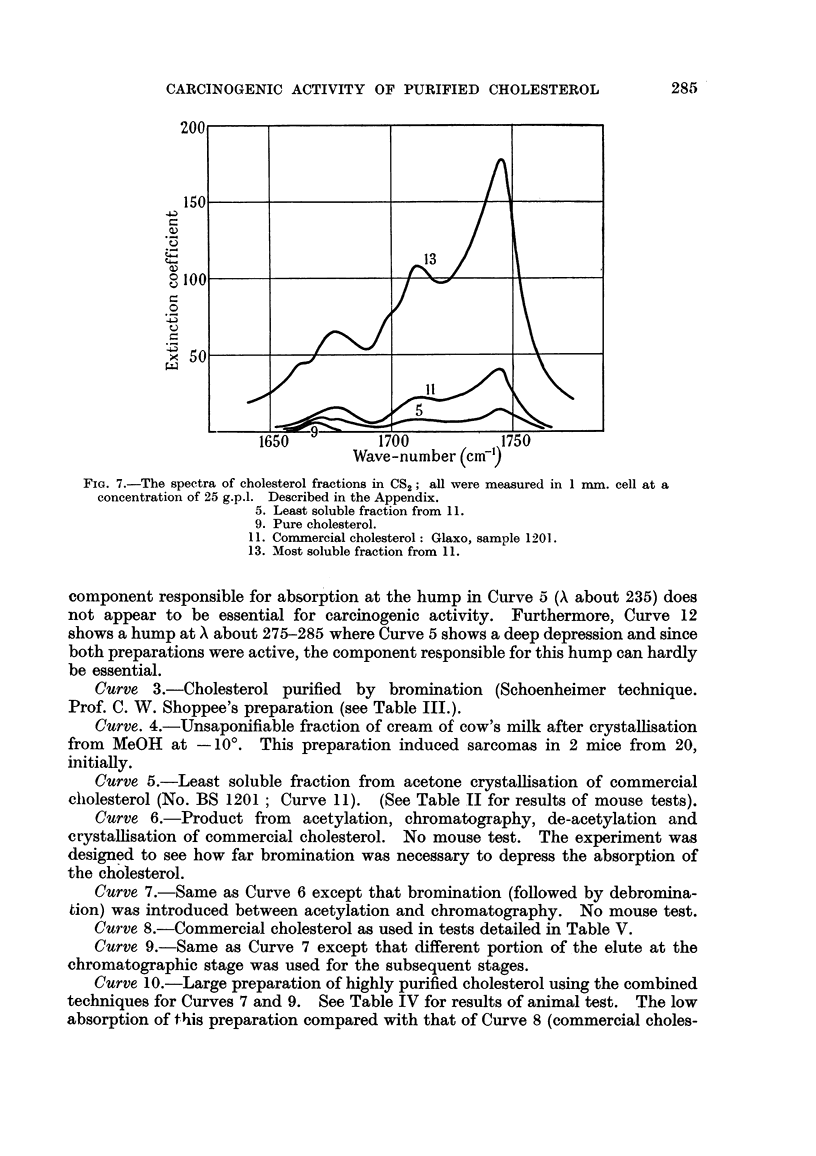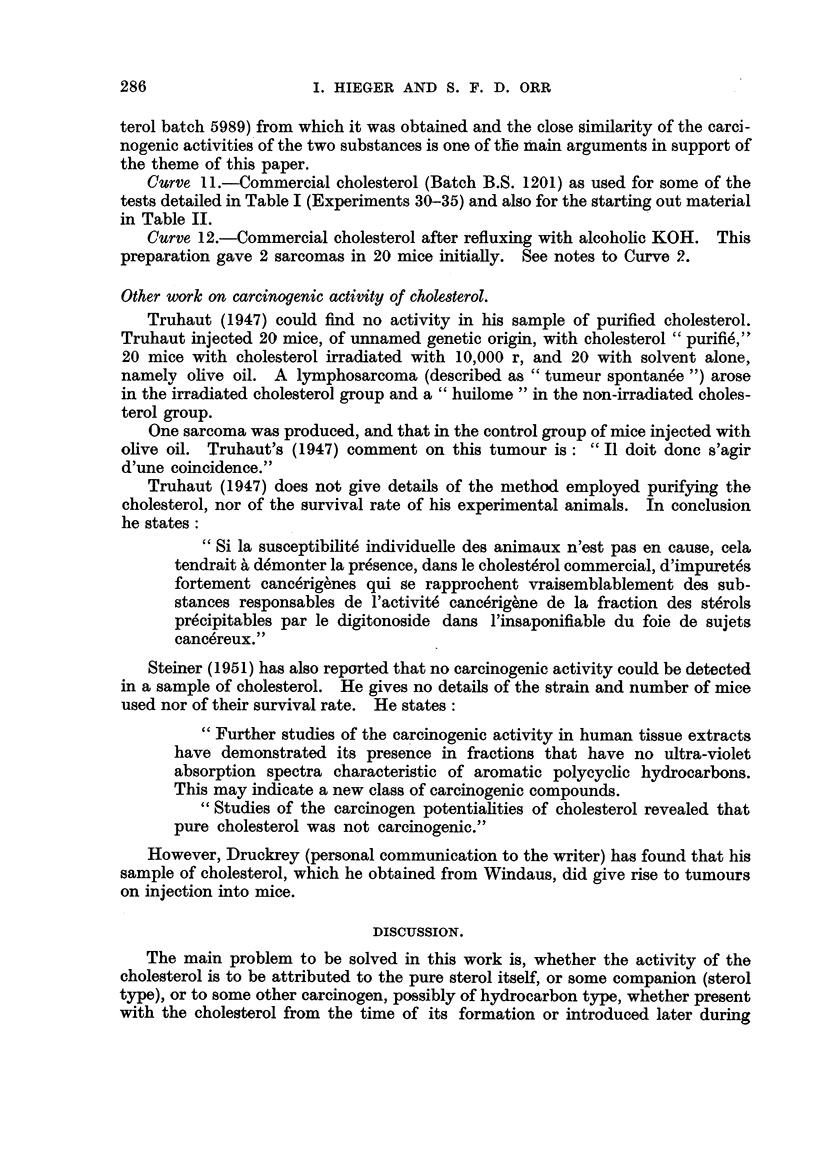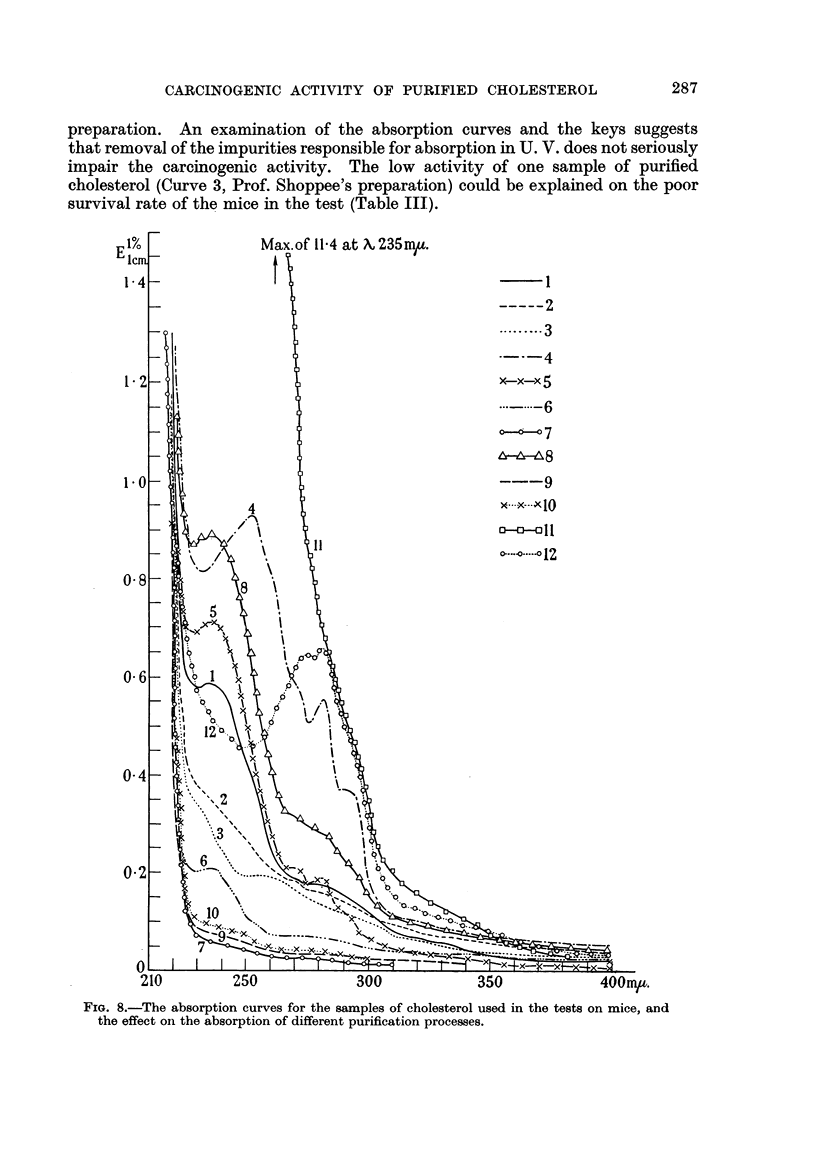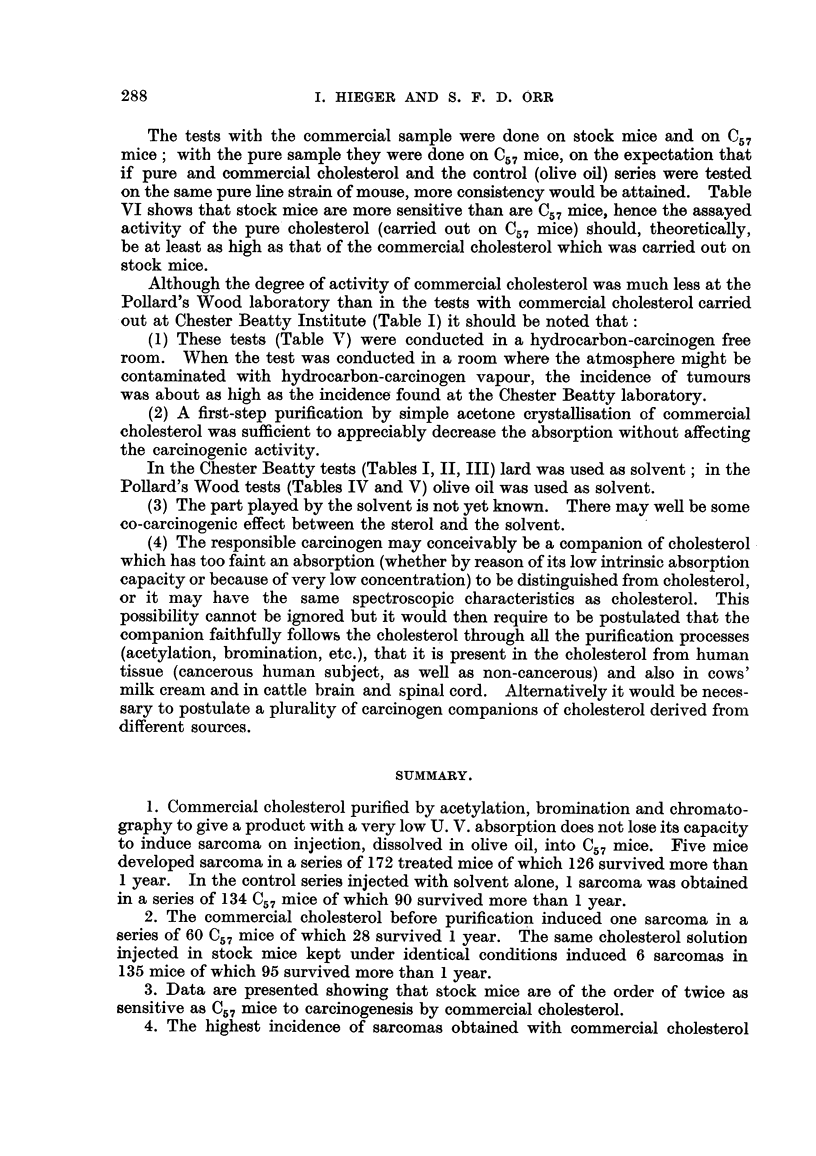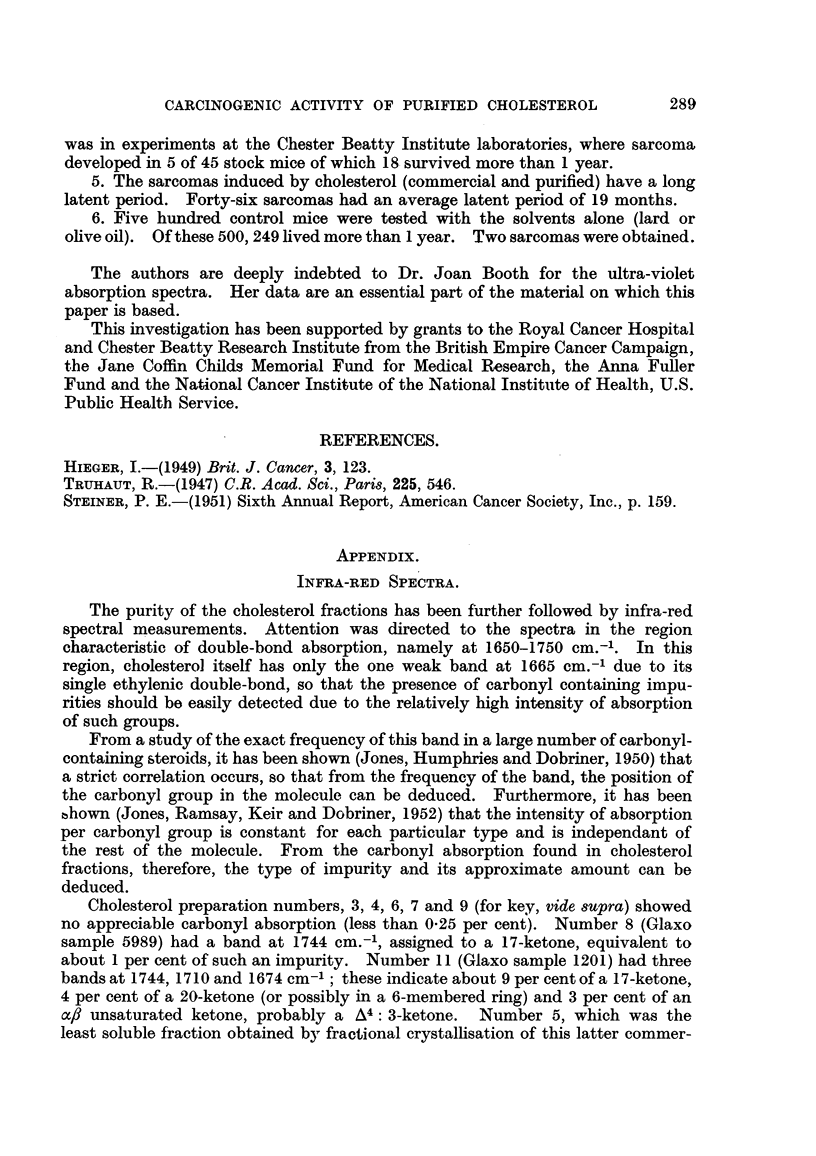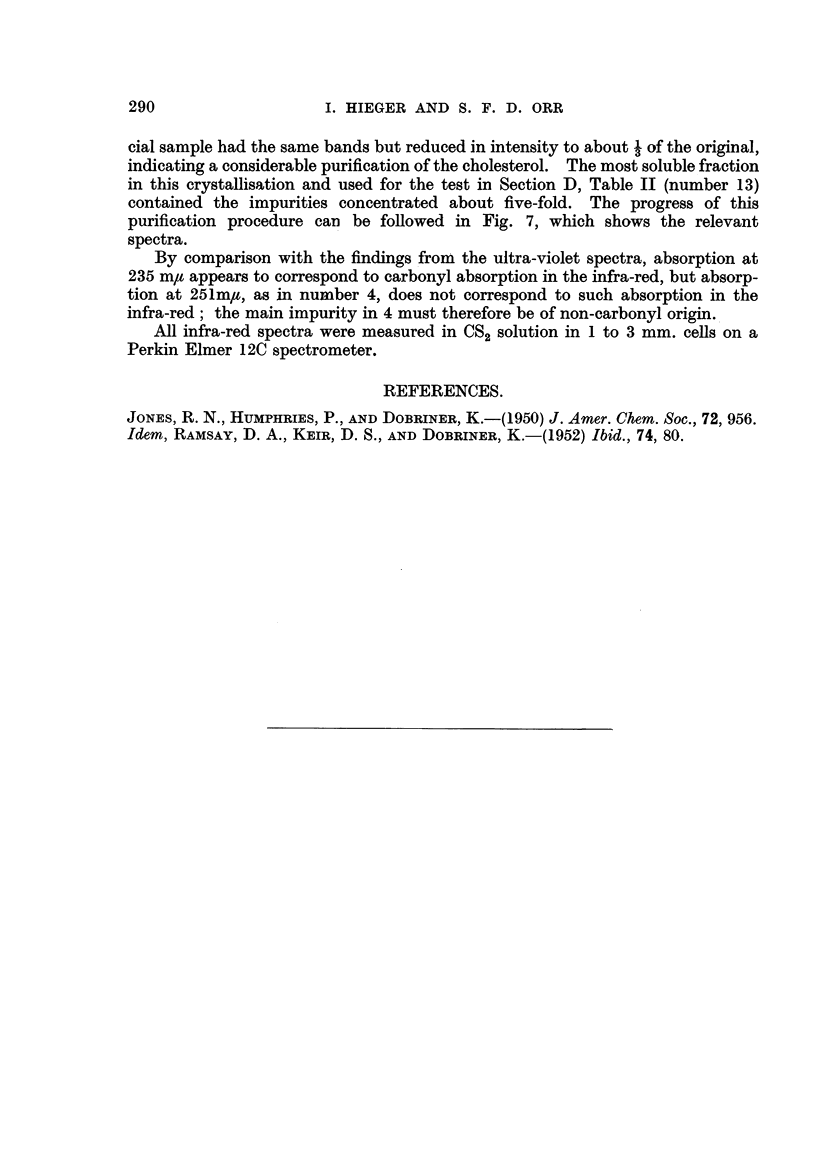# On the Carcinogenic Activity of Purified Cholesterol

**DOI:** 10.1038/bjc.1954.28

**Published:** 1954-06

**Authors:** I. Hieger, S. F. D. Orr

## Abstract

**Images:**


### ON THE CARCINOGENIC ACTIVITY OF PURIFIED CHOLESTEROL.

I. HIEGER AND S. F. D. ORR.

*From the Chester Beatty Research Institute, Institute of Cancer Research, Royal Cancer Hospital, London, S.W.3.*

Received for publication April 3, 1954.

THE development of the search for endogenic carcinogens has been reported in earlier publications from this Institute, (see Hieger (1949) for references to previous papers). It has been shown that the cholesterol-rich fraction of tissues from human subjects who had died of cancer or of other causes, and even commercial cholesterol, is a carcinogen. The present paper deals with the evidence for the carcinogenic activity of the purified sterol itself.

It will be useful to first present in greater detail the data for the results obtained with commercial cholesterol before it has undergone any special purification, i.e., as bought. Table I and Summary to Table I show the survival rate and tumour incidence in the first series of tests.

Some of the experiments in Table I have been reported in the 1949 paper (Hieger, 1949, p. 132, section 10*d*). They are repeated here especially to show that when this particular series of test was extended (it was carried on over the period 1944–1949) to further groups of mice, and, moreover, with different samples of commercial cholesterol, the activity of the sterol remained within the limits of uncertainty characteristic of the tests with this substance, provided other conditions remain unaltered.

All " tumours " in this paper are sarcomas at the site of injection, usually of spindle cell type. Photomicrographs are shown in the plates (Fig. 1 to 6). A good proportion of these sarcomas transplanted well. The first tumour in Table I was carried through 93 generations and the first tumour in Table IV (highly purified cholesterol) has been growing vigorously in its 25th generation. The latent period is in the neighbourhood of 19 months (Table IX). The technique of assay has been to inject 0·1–0·4 c.c. of a 10 or 15 per cent solution of the sterol subcutaneously into mice as often as is required to maintain a nodule under the skin. On the average, each mouse received of the order of 3 to 5 injections during the course of the test which lasted from the time the mice were 6–10 weeks of age until their death. In the earlier tests lard, supplied by a single firm, was used as the solvent, but in 1950 it was found that a large batch of lard from the same source had different physical properties from the lard supplied until then, and it was decided to change to olive oil as solvent, although that alteration did not guarantee that there would result a greater uniformity in the composition of the solvent at future times. It would have been advantageous from some points of view to have chosen a synthetic solvent such as tricaprylin, but since a naturally occurring fat, i.e., lard, had already been in use for a considerable time the change to another naturally occurring fat, olive oil, was considered not to be as drastic a change in technique as would changing to a synthetic solvent. The control tests on solvents are described further on in this paper.

Purification of the cholesterol was next undertaken, as a first step using the simplest technique. Table II gives the results of tests on fractions of commercial cholesterol obtained by crystallisation from acetone. Some of these results were given in a condensed form in the 1949 paper (Hieger, 1949) referred to above; they are repeated here so that the results for the different fractions can be compared.

TABLE I.—*Tests of Carcinogenic Activity of Commercial Cholesterol (Solution in Lard).*

This table is laid out in detail in order to show the expectedly discrete and spasmodic development of tumours which appear at a low incidence in a large series of animals.

Sarcoma development at site of injection is shown by a number in parenthesis indicating the latent period in months.

Each line represents the events occurring in the same cage.

| Expt. No. | Strain of mouse. | Number of mice surviving. Start of expt. | 6 m. | 9 m. | 1 yr. | 15 m. | 18 m. | 21 m. | 24 m. | 27 m. | 30 m. | Sarcomas. |
|---|---|---|---|---|---|---|---|---|---|---|---|---|
| 1 | $C_{57}$ ♂ | 5 | 5 | 4 | $^{11}1$ | 1 | 0 | – | – | – | – | 1 |
| 2 | $C_{57}$ | 5 | 5 | 5 | 0 | – | – | – | – | – | – | 0 |
| 3 | $C_{57}$ | 6 | 3 | 3 | 3 | 3 | 3 | 3 | $^{24}0$ | – | – | 1 |
| 4 | $C_{57}$ | 6 | 5 | 4 | 4 | 3 | 0 | – | – | – | – | 0 |
| 5 | CBA ♀ | 5 | 4 | 4 | 3 | 2 | 0 | – | – | – | – | 0 |
| 6 | CBA ♂ | 5 | 5 | 5 | 5 | 5 | 2 | 1 | 0 | – | – | 0 |
| 7 | $C_{57}$ ♀ | 5 | 5 | 5 | $5^{13}$ | 4 | 4 | 1 | 0 | – | – | 1 |
| 8 | $C_{57}$ | 6 | 4 | 0 | – | – | – | – | – | – | – | 0 |
| 9 | $C_{57}$ | 5 | 5 | 5 | 0 | – | – | – | – | – | – | 0 |
| 10 | $C_{57}$ ♀ | 5 | 5 | 5 | 5 | 5 | $2^{20}$ | $1^{22}$ | 0 | – | – | 2 |
| 11 | $C_3H$ ♂ | 5 | 5 | 4 | 2 | 1 | 0 | – | – | – | – | 0 |
| 12 | $C_3H$ ♂ | 5 | 3 | 3 | 2 | 0 | – | – | – | – | – | 0 |
| 13 | $C_{57}$ ♂ | 5 | 4 | 4 | 4 | 4 | 4 | 0 | – | – | – | 0 |
| 14 | Stock ♂ | 5 | 0 | – | – | – | – | – | – | – | – | 0 |
| 15 | ,, ♂ | 5 | 3 | 3 | 3 | 2 | 0 | – | – | – | – | 0 |
| 16 | ,, ♀ | 5 | 5 | 5 | 5 | 2 | 2 | 1 | 0 | – | – | 0 |
| 17 | ,, ♀ | 5 | 3 | 1 | 0 | – | – | – | – | – | – | 0 |
| 18 | ,, ♀ | 5 | 4 | 4 | 4 | $^{14}2^{16}$ | 0 | – | – | – | – | 2 |
| 19 | ,, ♂ | 5 | 4 | 4 | 4 | $3^{15}$ | 2 | 1 | 0 | – | – | 1 |
| 20 | $C_{57}$ ♂ | 5 | 5 | 5 | 5 | 5 | 4 | 4 | 3 | 0 | – | 0 |
| 21 | $C_{57}$ ♂ | 5 | 4 | 4 | 4 | 4 | $2^{18}$ | 1 | 1 | 1 | 0 | 1 |
| 22 | $C_{57}$ | 3 | 0 | – | – | – | – | – | – | – | – | 0 |
| 23 | $C_{57}$ | 4 | 4 | 4 | 4 | 4 | 4 | 4 | 3 | 3 | 0 | 0 |
| 24 | $C_{57}$ | 4 | 4 | 4 | 4 | 4 | 4 | 4 | 2 | 0 | – | 0 |
| 25 | $C_{57}$ ♀ | 4 | 4 | 4 | 4 | 3 | 3 | $2^{23}$ | 0 | – | – | 1 |
| 26 | $C_{57}$ | 5 | 3 | 2 | 2 | 2 | 1 | 1 | 0 | – | – | 0 |
| 27 | $C_{57}$ | 5 | 5 | 5 | 4 | 3 | $2^{18}$ | 0 | – | – | – | 1 |
| 28 | $C_{57}$ | 5 | 5 | 4 | 4 | 3 | 1 | 1 | 0 | – | – | 0 |
| 29 | $C_{57}$ | 5 | 4 | 3 | 2 | 0 | – | – | – | – | – | 0 |
| 30 | $C_{57}$ ♂ | 5 | 5 | 5 | 5 | 5 | 5 | 5 | 4 | $2^{29}$ | 0 | 1 |
| 31 | $C_{57}$ ♂ | 5 | 4 | 4 | 4 | 4 | 4 | 3 | 0 | – | – | 0 |
| 32 | Stock ♂ | 5 | 5 | 2 | $1^{14}$ | 0 | – | – | – | – | – | 1 |
| 33 | ,, ♀ | 5 | 2 | 0 | – | – | – | – | – | – | – | 0 |
| 34 | ,, ♂ | 5 | 2 | 1 | 1 | 1 | 1 | 1 | $1^{24}$ | 0 | – | 1 |
| 35 | $C_{57}$ ♀ | 5 | 4 | 4 | 4 | 4 | 4 | $3^{23}$ | 2 | 0 | – | 1 |
| 36 | MRC ♀ | 5 | 5 | 3 | 3 | 3 | 3 | 2 | 0 | – | – | 0 |
| 37 | ,, ♂ | 5 | 5 | 4 | 4 | 3 | 2 | $2^{23}$ | 0 | – | – | 1 |
| 38 | ,, ♂ | 5 | 5 | 5 | 5 | 3 | 3 | $3^{23}$ | 2 | 0 | – | 1 |
| 39 | ,, ♂ | 5 | 4 | 4 | 4 | 0 | – | – | – | – | – | 0 |

### SUMMARY OF TABLE I.—*Carcinogenic Activity of Commercial Cholesterol (Solution in Lard).*

Attention is particularly drawn to columns 12 and 13. Obviously mice which die before they reach the latent period should strictly speaking not be entered as having been at risk. The figures in Column 12, therefore, give a lower apparent incidence than is in fact the true incidence.

| Strain of mouse. | Number of mice surviving. Start of expt. | 6 m. | 9 m. | 1 yr. | 15 m. | 18 m. | 21 m. | 24 m. | 27 m. | Number of sarcomas. | Incidence of sarcomas calculated per 100 effective mice at Start (%). | 1 yr. (%). |
|---|---|---|---|---|---|---|---|---|---|---|---|---|
| (1) | (2) | (3) | (4) | (5) | (6) | (7) | (8) | (9) | (10) | (11) | (12) | (13) |
| $C_{57}$ | 108 | 92 | 83 | 68 | 61 | 45 | 27 | 10 | 6 | 10 | 9·3 | 14 |
| Stock | 45 | 28 | 20 | 18 | 10 | 5 | 5 | 3 | 1 | 5 | 11 | 28 |
| $C^3H$ | 10 | 8 | 7 | 4 | 1 | 0 | — | — | — | 0 | 0 | — |
| MRC | 20 | 19 | 16 | 16 | 10 | 8 | 7 | 2 | 0 | 2 | 10 | 12 |
| CBA | 10 | 9 | 9 | 8 | 7 | 2 | 1 | 0 | - | 0 | 0 | — |

### TABLE II.

Activity of (1) fractions of commercial cholesterol obtained by crystallising from acetone, (2) impurities in the same sample of commercial cholesterol obtained in the mother liquors from the acetone crystallisation of the acetate. These impurities still contain some cholesterol.

| Expt. | Material tested. | Mouse strain. | Start of expt. | Number of mice surviving. 6 m. | 9 m. | 1 yr. | 15 m. | 18 m. | 21 m. | 24 m. | 27 m. | 30 m. | Sarcomas. | Latent periods (months). |
|---|---|---|---|---|---|---|---|---|---|---|---|---|---|---|
| A | Commercial cholesterol (Expts. 30–35 from Table I) This test was carried out during the same period as B, C, D, E below | $C_{57}$ | 15 | 13 | 13 | 13 | 13 | 13 | 11 | 6 | 2 | 0 | 2 | 23, 19 |
| | | Stock | 15 | 9 | 3 | 2 | 1 | 1 | 1 | 1 | 0 | — | 2 | 14, 24 |
| B | Least soluble fraction from acetone crystallisation of the cholesterol used for A | $C_{57}$ | 10 | 9 | 9 | 8 | 7 | 6 | 2 | 2 | 0 | — | 1 | 18 |
| | | Stock | 15 | 6 | 5 | 5 | 4 | 4 | 1 | 0 | - | — | 3 | 14, 18, 21 |
| C | Moderately soluble fraction from same fractionation process which gave B | $C_{57}$ | 11 | 4 | 4 | 4 | 4 | 4 | 3 | 3 | 0 | — | 0 | — |
| | | Stock | 16 | 13 | 13 | 11 | 7 | 5 | 2 | 2 | 0 | — | 1 | 15 |
| D | Mother liquors from B, C fractionation | $C_{57}$ | 12 | 10 | 8 | 7 | 7 | 5 | 3 | 3 | 1 | 0 | 0 | — |
| | | Stock | 15 | 8 | 5 | 5 | 5 | 5 | 2 | 1 | 0 | — | 0 | — |
| E | Impurities in the same cholesterol as used in A–D, removed in the mother liquors from acetone crystallisation of the acetate (Preparation by Prof. C. W. Shoppee.) | $C_{57}$ | 11 | 11 | 11 | 10 | 9 | 8 | 6 | 3 | 0 | - | 0 | — |
| | | Stock | 11 | 6 | 6 | 4 | 4 | 3 | 1 | 0 | - | - | 0 | — |

### EXPLANATION OF PLATES.

FIG. 1.—Sarcoma (No. 1) induced at site of injection in a $C_{57}$ mouse, by olive oil solution of highly purified cholesterol. × 90.

FIG. 2.—Sarcoma (No. 2) induced at site of injection in a $C_{57}$ mouse, by olive oil solution of highly purified cholesterol. × 90.

FIG. 3.—Sarcoma (No. 3) induced at site of injection in a $C_{57}$ mouse, by olive oil solution of highly purified cholesterol. × 90.

FIG. 4.—Sarcoma (No. 4) induced at site of injection in a $C_{57}$ mouse, by olive oil solution of highly purified cholesterol. × 90.

FIG. 5.—Sarcoma (No. 5) induced at site of injection in a $C_{57}$ mouse, by olive oil solution of highly purified cholesterol. × 90.

FIG. 6.—Sarcoma induced at site of injection in a $C_{57}$ mouse by olive oil (control test). × 90.

Heiger and Orr.

A sample of pure cholesterol was prepared by Professor Shoppee using the Schoenheimer technique (bromination). The results of the mouse tests are shown in Table III.

TABLE III.—*Activity of Purified Cholesterol (prepared by Prof. C. W. Shoppee).*

| Strain of mouse. | Number of mice surviving. Start of expt. | 6 m. | 9 m. | 1 yr. | 15 m. | 18 m. | 21 m. | 24 m. | 27 m. | 30 m. | Sarcomas. |
|---|---|---|---|---|---|---|---|---|---|---|---|
| Stock ♀ | 5 | 4 | 4 | 4 | 4 | 3 | 2 | 2 | 1 | 0 | 1 (27th month) |
| ,, ♂ | 9 | 5 | 4 | 4 | 4 | 2 | 1 | 0 | – | – | |
| $C_{57}$ ♀ | 5 | 4 | 3 | 2 | 2 | 2 | 0 | – | – | – | – |
| ,, ♂ | 5 | 4 | 0 | 0 | – | – | – | – | – | – | – |
| ,, ♂ | 5 | 4 | 5 | 5 | 5 | 4 | 4 | 0 | – | – | – |
| ,, ♂ | 5 | 3 | 3 | 3 | 3 | 0 | – | – | – | – | – |

Cholesterol was next purified on a large scale. The method involved several stages. These were :

(1) Acetylation. This step provided several advantages, namely—
  (*a*) the OH group was protected during bromination ;
  (*b*) the acetate can be differentially eluted from $Al_2O_3$ by mild eluents : the unacetylated sterol when absorbed on $Al_2O_3$ requires more powerful eluents.
(2) Bromination, filtration and debromination.
(3) Adsorption on $Al_2O_3$ column and elution with benzene-pet. ether mixture.
(4) De-acetylation of the eluate by a mild saponifying agent ($KHCO_3$).
(5) Crystallisation from acetone.

The results of the mouse tests on this purified cholesterol are shown in Table IV. This experiment was carried out at our Buckinghamshire laboratories in the " Hydrocarbon-Carcinogen free laboratory "; the mice were housed in the same room as the control series (Table V) treated with the cholesterol before it had undergone purification.

*Atmospheric carcinogen as a possible factor in the induction of subcutaneous sarcomas*

The investigations were continued after 1949 at our country laboratories (at Pollards Wood, Buckinghamshire), which are situated 20 miles from London and could give an opportunity for carrying out this kind of work in an atmosphere probably less contaminated by carcinogens than at the Chester Beatty Institute in London where carcinogens are frequently used in different parts of the building. Experiments at Pollards Wood are conducted in rooms which have never, intentionally, contained carcinogens other than those of the sterol type. These laboratories are reached over an open court-yard, 20 yards away from other mouse rooms (referred to below as " carcinogen room "), where the mice injected with cholesterol are kept in boxes on the same racks as mice which are being treated by painting with potent hydrocarbon-type carcinogens (3 : 4-benzpyrene or dimethylbenzanthracene). By this arrangement it was possible to carry out experiments designed to test the effect of atmospheric contamination by powerful carcinogens.

There was, of course, no guarantee that contamination could have been completely avoided in the "carcinogen-free" room; for example, sawdust from a box containing benzpyrene-treated mice might accidentally be carried in to the non-carcinogen room on the shoes of anyone who had walked through the carcinogen room. However, the technicians who serviced the mice in the carcinogen room

TABLE IV.—*Activity of olive oil solution of highly purified cholesterol: Tests on $C_{57}$ mice.*

Sarcoma development at site of injection is indicated by a number in parentheses showing the latent period in months.

Experiments at Pollard's Wood laboratory.

| Start of expt. | Number of mice surviving. 6 m. | 9 m. | 1 yr. | 15 m. | 18 m. | 21 m. | 24 m. | 27 m. | 30 m. | Sarcomas. |
|---|---|---|---|---|---|---|---|---|---|---|
| 3 | 3 | 3 | 3 | 1 | 1 | 1 | 1 | 0 | – | 0 |
| 3 | 3 | 3 | 3 | 3 | 0 | – | – | – | – | 0 |
| 3 | 3 | 3 | 3 | 3 | 3 | $^{20}2$ | 0 | – | – | 1 ($C_{57}$ ♀) |
| 3 | 3 | 3 | 3 | 2 | 2 | 1 | 1 | 0 | – | 0 |
| 5 | 4 | 2 | 0 | – | – | – | – | – | – | 0 |
| 3 | 2 | 2 | 2 | 2 | 1 | 1 | 1 | 0 | – | 0 |
| 3 | 3 | 2 | 2 | 1 | 1 | 1 | 0 | – | – | 0 |
| 3 | 3 | 2 | 2 | 0 | – | – | – | – | – | 0 |
| 4 | 3 | 3 | 3 | 2 | 2 | 0 | – | – | – | 0 |
| 3 | 3 | 3 | 2 | 2 | 2 | 0 | – | – | – | 0 |
| 4 | 3 | 3 | 3 | 3 | 3 | $1^{23}$ | 0 | – | – | 1 ($C_{57}$ ♀) |
| 4 | 4 | 2 | 1 | 1 | 0 | – | – | – | – | 0 |
| 5 | 5 | 4 | 4 | 4 | 4 | 0 | – | – | – | 0 |
| 5 | 3 | 2 | 2 | 2 | 2 | 1 | 0 | – | – | 0 |
| 4 | 4 | 4 | 4 | 4 | 4 | 2 | 1 | 0 | – | 0 |
| 4 | 4 | 4 | 4 | 4 | 4 | 3 | 0 | – | – | 0 |
| 3 | 2 | 2 | 2 | 2 | 2 | 0 | – | – | – | 0 |
| 4 | 2 | 2 | 2 | 2 | 1 | 0 | – | – | – | 0 |
| 3 | 3 | 3 | 3 | 3 | 3 | 3 | 0 | – | – | 0 |
| 3 | 3 | 3 | 3 | $2^{15}$ | 1 | 1 | 0 | – | – | 1 ($C_{57}$ ♀) |
| 4 | 4 | 4 | 4 | 4 | 4 | 2 | 2 | – | – | 0 |
| 3 | 3 | 2 | 2 | 2 | 2 | 2 | 1 | 1 | 0 | 0 |
| 5 | 5 | 5 | 5 | 3 | 0 | – | – | – | – | 0 |
| 4 | 4 | 4 | 4 | 4 | 2 | 2 | 1 | 0 | – | 0 |
| 5 | 3 | 3 | 3 | 3 | 3 | 2 | 0 | – | – | 0 |
| 3 | 3 | 3 | 2 | 2 | 2 | 2 | 0 | – | – | 0 |
| 4 | 4 | 4 | 4 | 4 | 4 | 2 | 1 | 0 | – | 0 |
| 4 | 4 | 4 | 4 | 3 | 2 | 2 | 0 | – | – | 0 |
| 4 | 2 | 1 | 1 | 1 | 0 | – | – | – | – | 0 |
| 3 | 2 | 2 | 2 | 2 | $2^{18}$ | 1 | 0 | – | – | 1 ($C_{57}$ ♀) |
| 4 | 2 | 2 | 2 | 2 | 2 | 2 | 1 | 0 | – | 0 |
| 4 | 4 | 4 | 3 | 2 | 0 | – | – | – | – | 0 |
| 4 | 2 | 2 | 2 | 2 | 0 | – | – | – | – | 0 |
| 4 | 4 | 4 | 4 | 4 | 1 | 1 | 1 | 0 | – | 0 |
| 5 | 5 | 5 | 5 | 5 | 4 | 4 | 3 | 2 | 1 | 0 |
| 4 | 4 | 2 | 2 | 2 | 2 | 2 | 0 | – | – | 0 |
| 4 | 3 | 3 | 3 | 3 | $3^{19}$ | 1 | 0 | – | – | 1 ($C_{57}$ ♂) |
| 4 | 2 | 2 | 2 | 1 | 0 | – | – | – | – | 0 |
| 6 | 5 | 5 | 5 | 4 | 2 | 0 | – | – | – | 0 |
| 5 | 5 | 4 | 3 | 2 | 2 | 2 | 2 | 0 | – | 0 |
| 4 | 4 | 4 | 4 | 4 | 2 | 1 | 1 | 0 | – | 0 |
| 5 | 5 | 4 | 4 | 4 | 3 | 3 | 1 | 0 | – | 0 |
| 5 | 5 | 5 | 4 | 4 | 3 | 2 | 2 | 1 | – | – |
| 3 | 2 | 2 | 1 | 0 | – | – | – | – | – | – |
| 172 | 149 | 135 | 126 | 110 | 81 | 50 | 20 | 4 | 1 | 5 |

were only allowed to handle the mice in the non-carcinogen room during a fortnight (vacation) per year, when the " non-carcinogen " technician was not available.

More complete isolation of the " non-carcinogen " experiment could have been achieved by having a self-contained unit, rooms, equipment, servicing and staff completely separate from the main block of laboratories.

The results of the tests are shown in Table V.

The findings summarised in the foregoing tables suggest answers to a number of questions on the subject of this paper. For example :

*Injury* per se, *i.e., the pricking of the tissue on injection, as a possible factor in sarcoma production.*

The mechanical trauma caused by the needle cannot be an important factor for only 1 sarcoma appeared in over 360 mice, repeatedly injected with lard alone (control) in one series of tests and only 1 in another control series of 134 mice injected with olive oil alone. Moreover, the fatty solvent is more rapidly dispersed in the subcutaneous tissue than it is when loaded with 10–15 per cent of sterol and consequently the controls had more injections than the sterol injected mice in order to maintain a stationary nodule.

*Is the carcinogenic activity of commercial cholesterol due* (a) *to the sterol itself, or* (b) *to highly active impurities ?*

The evidence presented in Tables IV and V provides support for (*a*), i.e., cholesterol *per se* is the true carcinogen in the commercial product.

If the incidence of sarcomas be expressed as the ratio (percentage) of tumour mice to injected mice which survived to a minimal latent period level, say 1 year, then the result can be stated thus : (*a*) Purified cholesterol gives rise to 4 per cent sarcomas in $C_{57}$ mice, (*b*) the cholesterol before purification gave 6·4 per cent (average of 7·9 and 5·4 per cent) in stock mice ; in $C_{57}$ mice the yield was 3·6 per cent but as this figure depends upon one solitary sarcoma it would not be reliable except for the striking fact that in a number of tests $C_{57}$ mice have repeatedly proved to be about half as sensitive to cholesterol as are stock mice (Table VI).

To assess the carcinogenic activity of a material by a number denoting the ratio (as a percentage) of tumour bearing mice to the total number of mice treated cannot be satisfactory, for obviously the mice which die before the average expected latent period cannot have had adequate exposure to the carcinogen. It would be an improvement if the assay were calculated as the percentage of tumours induced in the mice which survive to the average latent period or at least to the minimum latent period.

The data obtained from the experiments carried out with purified sterol at the Chester Beatty Institute (Table III) do not support the contention that the carcinogenesis is due to the sterol, but it should be observed that some disturbing factors appeared in this test. The mice did not survive well, and in fact the single sarcoma appeared at the 27th month in the sole survivor of the 34 mice initially injected with purified cholesterol. Twenty-seven months is near the outside limit of the range of latent periods found with the carcinogens of the cholesterol category. In an experiment with commercial cholesterol (Table II, Section A) 3 of the 4

TABLE V.—*Tumour Induction by Olive Oil Solution of Commercial Cholesterol, and of Highly Purified Cholesterol.*

Experiments at Pollards Wood laboratory.

| Expt. | Mouse strain. | Location. | Number of mice at start. | Number of mice surviving. 6 m. | 9 m. | 1 yr. | 15 m. | 18 m. | 21 m. | 24 m. | 27 m. | 30 m. | 33 m. | Number of sarcomas. | Sarcoma incidence calculated per 100 mice: At start (%) | At 1 yr. (%) | Latent period of sarcomas (months). |
|---|---|---|---|---|---|---|---|---|---|---|---|---|---|---|---|---|---|
| A | $C_{57}$ | Hydrocarbon-carcinogen free laboratory | 60 | 34 | 30 | 28 | 21 | 14 | 9 | 3 | 1 | — | — | 1 | 1·7 | 3·6 | 22 |
| B | Stock | Ditto | 70 | 45 | 38 | 38 | 22 | 18 | 10 | 2 | 0 | — | — | 3 | 4·3 | 7·9 | 12, 16, 24 |
| C | ,, | ,, | 65 | 63 | 62 | 57 | 55 | 41 | 13 | 11 | 6 | 0 | — | 3 | 4·6 | 5·4 | 20, 20, 29 |
| D | ,, | Carcinogen laboratory* | 100 | — | — | 75 | 64 | 45 | 32 | 10 | 2 | — | — | 8 | 8 | 10·7 | 11, 12, 16, 16, 17, 20, 20, 24 |
| E | CBA | Carcinogen laboratory* | 100 | — | — | 71 | 47 | 45 | 40 | 29 | 18 | 5 | 0 | 2 | 2 | 2·8 | 19, 27 |
| (For comparison) | SUMMARY OF TABLE IV (Highly purified cholesterol). | | | | | | | | | | | | | | | | |
| F (Table IV) | $C_{57}$ | Hydrocarbon-carcinogen free laboratory | 172 | 149 | 135 | 126 | 110 | 81 | 50 | 20 | 4 | 0 | — | 5 | 2·9 | 4 | 15, 18, 19 20, 23 |

(These mice were housed in the same room and kept under the same conditions of diet, etc., as the mice in sections A, B, C above.)

* " Carcinogen laboratory." In this experiment the mice were housed in the same room and on the same racks as were mice which were being painted bi-wieekly with benzpyrene or with dimethylbenzanthracene in an attempt to produce a carcinogen laden atmosphere.

TABLE VI.—*To Illustrate the Higher Response of Stock Mice, as Compared with $C_{57}$ Mice, to the Carcinogenic Action of Cholesterol.*

The data are arranged in pairs ($C_{57}$ : Stock — $C_{57}$ : Stock — etc.). All the mice *within each pair* were injected with the same material, kept in the same room and fed the same diet.

| Table. | Strain. | Number of mice. | Survived 1 year. | Number of sarcomas. | Sarcomas per 100 mice initial (%) | Sarcomas per 100 mice surviving 1 yr. (%) |
|---|---|---|---|---|---|---|
| I | $C_{57}$ | 108 | 68 | 10 | 9·3 | 14 |
| | Stock | 45 | 18 | 5 | 11 | 28 |
| II, Section A | $C_{57}$ | 15 | 13 | 2 | 13 | 15 |
| | Stock | 15 | 2 | 2 | 13 | 100 |
| II, Section B | $C_{57}$ | 10 | 8 | 1 | 10 | 12 |
| | Stock | 15 | 5 | 3 | 20 | 60 |
| V, Sections A, B, C | $C_{57}$ | 60 | 28 | 1 | 1·7 | 3·6 |
| | Stock | 135 | 95 | 6 | 4·4 | 6·3 |

sarcomas had latent periods of 23, 24 and 29 months respectively. In the test just discussed (Table III) only 3 mice survived to 21 months, 2 to 24 months and 1 to 27 months when it developed a sarcoma. It is quite possible that the susceptible mice in this group died before the characteristic latent period had elapsed. Table IX shows the distribution of latent periods of the sarcomas discussed in this paper.

Why one mouse should develop a sarcoma while others of the same batch of similar stock or strain treated under the same conditions should not, has still to be explained. To say that the single positive mouse had the necessary degree of susceptibility does not, of course, advance the understanding of the difference. It is highly probable that multiple factors are necessary for sarcoma induction with this type of carcinogen. Seven suggest themselves, namely : susceptibility associated with strain, sex and the individual animal, environmental conditions such as atmospheric and dietetic, the " true " potency of the carcinogen and the nature of the vehicle for administration.

*The effect of strain differences on susceptibility.*

The summary of Table I, Table V and Table VI illustrates the effect on tumour incidence of varying the strain of mouse while keeping other factors the same. Table VI particularly shows that although tumour yield varied, the relative order of susceptibility of stock mice and of $C_{57}$ mice remained substantially the same.

*Could the injected cholesterol act as a reservoir for accumulating any circulating carcinogen, either of endogenic or of exogenic origin?*

This possibility, which was put forward first by Kennaway in 1936 (unpublished) and by J. W. Orr in 1947 (unpublished), would provide an attractive theory of the mode of action of cholesterol as carcinogen. However, it would be necessary to postulate that the carcinogen is held by adsorption on the cholesterol although still in an active stage, and that in such a condition it is not readily eluted, for clearly if it were a case of simple solubility there would be no more reason for the factor dissolving in the cholesterol than for it to be dissolved out again. Secondly injected cholesterol after a year or so in the body should be rich in absorbed carcinogen. Thirdly, if such an assumption were accepted, there would be little necessity to proceed with the question of whether pure or impure cholesterol is the true carcinogen. Fourthly, if the carcinogen which is concentrated in the cholesterol originated in the environment, it should be easy to test the hypothesis by purposely allowing the mice injected with the cholesterol to live in an atmosphere rich in carcinogen.

This test has been put into operation, and constitutes a second stage to the investigation on atmospheric carcinogens as a factor in sarcoma induction (see above).

One hundred stock mice were injected with olive oil solution of commercial cholesterol and kept in a carcinogen-rich atmosphere as described above. The control mice (135) were kept in the room free from carcinogen.

The results are shown in Table V.

*Control tests on the solvents.*

Lard was employed as solvent from 1936 to 1949. During this time a total of over 360 mice was tested with the solvent, lard, alone. These mice did not survive remarkably well; they gave no positive result until the series had been almost completed, when among the last few batches a single sarcoma developed. When in 1950, a change was made from lard to olive oil (hospital dispensary grade), a control series of 134 mice was set up for injection with the oil alone. A single sarcoma was found in this series. A detailed analysis of these tests is shown in Tables VII and VIII.

For the experiments described in Tables I, II, and III and Flow Sheet I lard was used as solvent. In the other experiments olive oil was used.

TABLE VII.—*Control Tests on Solvent Alone (Lard).*

One sarcoma only was obtained in these control tests on 366 mice. The "old $C_{57}$" mice were 12 (and upwards) months of age at the start of the experiments; their effective number should therefore be raised by a factor of the order of 2 since the initial number, 19, would represent the survivors at 12 months. The single sarcoma occurred in an old $C_{57}$ mouse at the unexpectedly low latent period of 6 months.* However, since this mouse was 16 months of age at the start of the experiment some ageing factor for tumour induction is obviously operating. When young mice are used, the average latent period is 19 months (Table IX).

| | Number of mice at start and survivors. | | | | | | | | |
|---|---|---|---|---|---|---|---|---|---|
| | At 1st injection. | 6 months. | 9 months. | 12 months. | 15 months. | 18 months. | 21 months. | 24 months. | 27 months. |
| Stock | 307 | 226 | 179 | 129 | 89 | 56 | 27 | 13 | 2 |
| $C_{57}$ | 40 | 31 | 30 | 26 | 22 | 22 | 10 | 3 | — |
| $C_{57}$ (old) | 19 | 13* | 9 | 4 | 4 | 3 | — | — | — |

### TABLE VIII.—*Tests on Activity of Solvent (Olive Oil) Alone).* *($C_{57}$ mice were used.)*

Experiments at Pollard's Wood in hydrocarbon-carcinogen free laboratory.

| Start of expt. | Number of mice surviving. | | | | | | | | Sarcomas. |
|---|---|---|---|---|---|---|---|---|---|
| | 6 m. | 9 m. | 1 yr. | 15 m. | 18 m. | 21 m. | 24 m. | 27 m. | |
| 4 | 4 | 4 | 4 | 2 | 1 | 0 | – | – | – |
| 5 | 5 | 5 | 4 | 2 | 2 | 2 | 0 | – | – |
| 4 | 4 | 4 | 4 | 4 | 2 | 2 | 1 | – | – |
| 4 | 4 | 4 | 3 | 2 | 2 | 1 | 0 | – | – |
| 5 | 4 | 4 | 4 | 4 | 0 | – | – | – | – |
| 3 | 2 | 2 | 2 | 2 | 1 | 0 | – | – | – |
| 3 | 3 | 3 | 3 | 3 | 2 | 1 | 0 | – | – |
| 3 | 2 | 2 | 2 | 2 | 2 | 0 | – | – | – |
| 3 | 3 | 3 | 3 | 1 | 1 | 1 | – | – | – |
| 4 | 4 | 3 | 3 | 3 | 0 | – | – | – | – |
| 4 | 3 | 3 | 3 | 3 | 3 | 2 | 0 | – | – |
| 3 | 1 | 0 | – | – | – | – | – | – | – |
| 4 | 2 | 2 | 2 | 2 | 2 | 2 | 0 | – | – |
| 4 | 3 | 2 | 2 | 2 | 2 | 0 | – | – | – |
| 3 | 3 | 3 | 3 | 3 | 0 | – | – | – | – |
| 3 | 2 | 2 | 2 | 2 | 2 | 1 | 0 | – | – |
| 3 | 2 | 2 | 2 | 2 | 2 | 1 | 0 | – | – |
| 3 | 3 | 3 | 3 | 3 | 3 | 2 | 0 | – | – |
| 3 | 3 | 1 | 0 | – | – | – | – | – | – |
| 4 | 4 | 4 | 4 | 4 | 3 | 3 | 0 | – | – |
| 3 | 3 | 3 | 3 | 3 | 2 | 2 | 2 | 2 | – |
| 6 | 2 | 2 | 2 | 2 | 2 | 2 | 2 | – | – |
| 6 | 6 | 5 | 3 | 2 | 2 | 2 | 0 | – | 1 ($C_{57}$ ♀) at 21st mth. |
| 5 | 5 | 4 | 4 | 4 | 2 | 0 | – | – | – |
| 6 | 4 | 4 | 4 | 4 | 0 | – | – | – | – |
| 6 | 4 | 4 | 4 | 3 | 1 | 0 | – | – | – |
| 6 | 6 | 5 | 5 | 3 | 2 | 0 | – | – | – |
| 6 | 4 | 4 | 4 | 4 | 1 | 1 | 1 | – | – |
| 6 | 2 | 1 | 1 | 1 | 1 | 0 | – | – | – |
| 6 | 5 | 4 | 3 | 3 | 2 | 0 | – | – | – |
| 6 | 5 | 3 | 4 | 2 | 0 | – | – | – | – |
| 134 | 107 | 95 | 90 | 77 | 45 | 25 | 6 | 2 | |

### TABLE IX—*Latent Periods of Sarcomas Induced with Cholesterol of Different Degrees of Purity.*

| Table No. | Number of sarcomas. | Latent periods (months). | Mean latent period. |
|---|---|---|---|
| I (including Section A) of Table II) | 17 | 11, 13, 14, 14, 15, 16, 18, 18, 20, 22, 22, 23, 23, 23, 24, 24, 29 | 19 |
| II (B and C) | 5 | 14, 15, 18, 18, 21 | 17 |
| III | 1 | 27 | 27 |
| IV | 5 | 15, 18, 19, 20, 23 | 19 |
| V (excluding Section F which is detailed in IV above) | 17 | 11, 12, 12, 16, 16, 16, 17, 19, 20, 20, 20, 20, 22, 24, 24, 27, 29 | 19 |
| VIII | 1 | 21 | 21 |
| | 46 | | |

*U.V. Spectroscopic Examination of Cholesterol of Various Degrees of Purity.*

*Key to Absorption Spectrum Curves* (*Fig.* 8).

*Curve* 1.—*Cholesterol* purified via dibromide and recrystallised from acetic acid. (Not tested on mice).

*Curve* 2.—*Acetone crystallised fraction of cholesterol fraction from liver* Stage *i*, below). Liver, obtained post mortem from human subjects who had died of cancer or from some other cause was saponified and treated according to the flow sheet below.

FLOW SHEET I.

| Fraction | | Mice at start. | After 1 yr. | Sarcomas. |
|---|---|---|---|---|
| Unsaponified fraction of human liver (partly cancer subjects, partly non-cancer) | *a* | 15 | 12 | 0 |
| ↓ resaponified and extracted with boiling MeOH | | | | |
| MeOH insoluble | *b* | 15 | 12 | 0 |
| MeOH moderately soluble | *c* | 10 | 8 | 0 |
| MeOH soluble filtered at — 10' | | | | |
| mother liquors | *d* | 15 | 12 | 0 |
| MeOH, -10° crystals | *e* | 15 | 12 | 0 |
| resaponified and crystallised from hot MeOH | | | | |
| mother liquors | *f* | 10 | 10 | 0 |
| MeOH, hot, crystals | *g* | 10 | 8 | 1 |
| crystallised from acetone | | | | |
| mother liquors | *h* | 5 | 4 | 1 |
| acetone, crystals | *i* | 15 | 14 | 0 |

The low absorption shown by Curve 2 (low, that is, compared with the curve for the commercial product) and its inactivity, carcinogenically, suggests that treatment by crystallising from acetone removes the active fraction. However, it is difficult to explain why Fractions *a* and *e* which contained all the subsequent fractions, including *i*, did not induce tumours. When this process (crystallising from acetone) was applied on a larger scale (Table II) to commercial cholesterol it was found that the active fraction was concentrated in the crystalline product which was much more active than the mother liquors, and here the absorption curve of the acetone crystal fraction (Curve 5) showed considerably less absorption than did the cholesterol before treatment (Curve 11). Moreover, number 12 which shows a depression where Curve 5 shows a hump is the absorption curve of a carcinogenically active preparation, (2 tumours in 20 mice (initially) using cholesterol refluxed with alcoholic KOH in an experiment designed to test whether the process of saponification itself confers activity on cholesterol). Therefore, the

FIG. 7.—The spectra of cholesterol fractions in $CS_2$; all were measured in 1 mm. cell at a concentration of 25 g.p.l. Described in the Appendix.
5. Least soluble fraction from 11.
9. Pure cholesterol.
11. Commercial cholesterol: Glaxo, sample 1201.
13. Most soluble fraction from 11.

component responsible for absorption at the hump in Curve 5 ($\lambda$ about 235) does not appear to be essential for carcinogenic activity. Furthermore, Curve 12 shows a hump at $\lambda$ about 275–285 where Curve 5 shows a deep depression and since both preparations were active, the component responsible for this hump can hardly be essential.

*Curve* 3.—Cholesterol purified by bromination (Schoenheimer technique. Prof. C. W. Shoppee's preparation (see Table III.).

*Curve.* 4.—Unsaponifiable fraction of cream of cow's milk after crystallisation from MeOH at $-10°$. This preparation induced sarcomas in 2 mice from 20, initially.

*Curve* 5.—Least soluble fraction from acetone crystallisation of commercial cholesterol (No. BS 1201 ; Curve 11). (See Table II for results of mouse tests).

*Curve* 6.—Product from acetylation, chromatography, de-acetylation and crystallisation of commercial cholesterol. No mouse test. The experiment was designed to see how far bromination was necessary to depress the absorption of the cholesterol.

*Curve* 7.—Same as Curve 6 except that bromination (followed by debromination) was introduced between acetylation and chromatography. No mouse test.

*Curve* 8.—Commercial cholesterol as used in tests detailed in Table V.

*Curve* 9.—Same as Curve 7 except that different portion of the elute at the chromatographic stage was used for the subsequent stages.

*Curve* 10.—Large preparation of highly purified cholesterol using the combined techniques for Curves 7 and 9. See Table IV for results of animal test. The low absorption of this preparation compared with that of Curve 8 (commercial cholesterol batch 5989) from which it was obtained and the close similarity of the carcinogenic activities of the two substances is one of the main arguments in support of the theme of this paper.

*Curve* 11.—Commercial cholesterol (Batch B.S. 1201) as used for some of the tests detailed in Table I (Experiments 30–35) and also for the starting out material in Table II.

*Curve* 12.—Commercial cholesterol after refluxing with alcoholic KOH. This preparation gave 2 sarcomas in 20 mice initially. See notes to Curve 2.

*Other work on carcinogenic activity of cholesterol.*

Truhaut (1947) could find no activity in his sample of purified cholesterol. Truhaut injected 20 mice, of unnamed genetic origin, with cholesterol " purifié," 20 mice with cholesterol irradiated with 10,000 r, and 20 with solvent alone, namely olive oil. A lymphosarcoma (described as " tumeur spontanée ") arose in the irradiated cholesterol group and a " huilome " in the non-irradiated cholesterol group.

One sarcoma was produced, and that in the control group of mice injected with olive oil. Truhaut's (1947) comment on this tumour is : " Il doit donc s'agir d'une coincidence."

Truhaut (1947) does not give details of the method employed purifying the cholesterol, nor of the survival rate of his experimental animals. In conclusion he states :

> " Si la susceptibilité individuelle des animaux n'est pas en cause, cela tendrait à démonter la présence, dans le cholestérol commercial, d'impuretés fortement cancérigènes qui se rapprochent vraisemblablement des substances responsables de l'activité cancérigène de la fraction des stérols précipitables par le digitonoside dans l'insaponifiable du foie de sujets cancéreux."

Steiner (1951) has also reported that no carcinogenic activity could be detected in a sample of cholesterol. He gives no details of the strain and number of mice used nor of their survival rate. He states :

> " Further studies of the carcinogenic activity in human tissue extracts have demonstrated its presence in fractions that have no ultra-violet absorption spectra characteristic of aromatic polycyclic hydrocarbons. This may indicate a new class of carcinogenic compounds.
>
> " Studies of the carcinogen potentialities of cholesterol revealed that pure cholesterol was not carcinogenic."

However, Druckrey (personal communication to the writer) has found that his sample of cholesterol, which he obtained from Windaus, did give rise to tumours on injection into mice.

### DISCUSSION.

The main problem to be solved in this work is, whether the activity of the cholesterol is to be attributed to the pure sterol itself, or some companion (sterol type), or to some other carcinogen, possibly of hydrocarbon type, whether present with the cholesterol from the time of its formation or introduced later during preparation. An examination of the absorption curves and the keys suggests that removal of the impurities responsible for absorption in U. V. does not seriously impair the carcinogenic activity. The low activity of one sample of purified cholesterol (Curve 3, Prof. Shoppee's preparation) could be explained on the poor survival rate of the mice in the test (Table III).

FIG. 8.—The absorption curves for the samples of cholesterol used in the tests on mice, and the effect on the absorption of different purification processes.

The tests with the commercial sample were done on stock mice and on $C_{57}$ mice; with the pure sample they were done on $C_{57}$ mice, on the expectation that if pure and commercial cholesterol and the control (olive oil) series were tested on the same pure line strain of mouse, more consistency would be attained. Table VI shows that stock mice are more sensitive than are $C_{57}$ mice, hence the assayed activity of the pure cholesterol (carried out on $C_{57}$ mice) should, theoretically, be at least as high as that of the commercial cholesterol which was carried out on stock mice.

Although the degree of activity of commercial cholesterol was much less at the Pollard's Wood laboratory than in the tests with commercial cholesterol carried out at Chester Beatty Institute (Table I) it should be noted that:

(1) These tests (Table V) were conducted in a hydrocarbon-carcinogen free room. When the test was conducted in a room where the atmosphere might be contaminated with hydrocarbon-carcinogen vapour, the incidence of tumours was about as high as the incidence found at the Chester Beatty laboratory.

(2) A first-step purification by simple acetone crystallisation of commercial cholesterol was sufficient to appreciably decrease the absorption without affecting the carcinogenic activity.

In the Chester Beatty tests (Tables I, II, III) lard was used as solvent; in the Pollard's Wood tests (Tables IV and V) olive oil was used as solvent.

(3) The part played by the solvent is not yet known. There may well be some co-carcinogenic effect between the sterol and the solvent.

(4) The responsible carcinogen may conceivably be a companion of cholesterol which has too faint an absorption (whether by reason of its low intrinsic absorption capacity or because of very low concentration) to be distinguished from cholesterol, or it may have the same spectroscopic characteristics as cholesterol. This possibility cannot be ignored but it would then require to be postulated that the companion faithfully follows the cholesterol through all the purification processes (acetylation, bromination, etc.), that it is present in the cholesterol from human tissue (cancerous human subject, as well as non-cancerous) and also in cows' milk cream and in cattle brain and spinal cord. Alternatively it would be necessary to postulate a plurality of carcinogen companions of cholesterol derived from different sources.

### SUMMARY.

1. Commercial cholesterol purified by acetylation, bromination and chromatography to give a product with a very low U. V. absorption does not lose its capacity to induce sarcoma on injection, dissolved in olive oil, into $C_{57}$ mice. Five mice developed sarcoma in a series of 172 treated mice of which 126 survived more than 1 year. In the control series injected with solvent alone, 1 sarcoma was obtained in a series of 134 $C_{57}$ mice of which 90 survived more than 1 year.

2. The commercial cholesterol before purification induced one sarcoma in a series of 60 $C_{57}$ mice of which 28 survived 1 year. The same cholesterol solution injected in stock mice kept under identical conditions induced 6 sarcomas in 135 mice of which 95 survived more than 1 year.

3. Data are presented showing that stock mice are of the order of twice as sensitive as $C_{57}$ mice to carcinogenesis by commercial cholesterol.

4. The highest incidence of sarcomas obtained with commercial cholesterol was in experiments at the Chester Beatty Institute laboratories, where sarcoma developed in 5 of 45 stock mice of which 18 survived more than 1 year.

5. The sarcomas induced by cholesterol (commercial and purified) have a long latent period. Forty-six sarcomas had an average latent period of 19 months.

6. Five hundred control mice were tested with the solvents alone (lard or olive oil). Of these 500, 249 lived more than 1 year. Two sarcomas were obtained.

The authors are deeply indebted to Dr. Joan Booth for the ultra-violet absorption spectra. Her data are an essential part of the material on which this paper is based.

This investigation has been supported by grants to the Royal Cancer Hospital and Chester Beatty Research Institute from the British Empire Cancer Campaign, the Jane Coffin Childs Memorial Fund for Medical Research, the Anna Fuller Fund and the National Cancer Institute of the National Institute of Health, U.S. Public Health Service.

### REFERENCES.

Hieger, I.—(1949) *Brit. J. Cancer*, **3**, 123.
Truhaut, R.—(1947) *C.R. Acad. Sci., Paris*, **225**, 546.
Steiner, P. E.—(1951) Sixth Annual Report, American Cancer Society, Inc., p. 159.

### Appendix.

### Infra-red Spectra.

The purity of the cholesterol fractions has been further followed by infra-red spectral measurements. Attention was directed to the spectra in the region characteristic of double-bond absorption, namely at 1650–1750 cm.$^{-1}$. In this region, cholesterol itself has only the one weak band at 1665 cm.$^{-1}$ due to its single ethylenic double-bond, so that the presence of carbonyl containing impurities should be easily detected due to the relatively high intensity of absorption of such groups.

From a study of the exact frequency of this band in a large number of carbonyl-containing steroids, it has been shown (Jones, Humphries and Dobriner, 1950) that a strict correlation occurs, so that from the frequency of the band, the position of the carbonyl group in the molecule can be deduced. Furthermore, it has been shown (Jones, Ramsay, Keir and Dobriner, 1952) that the intensity of absorption per carbonyl group is constant for each particular type and is independant of the rest of the molecule. From the carbonyl absorption found in cholesterol fractions, therefore, the type of impurity and its approximate amount can be deduced.

Cholesterol preparation numbers, 3, 4, 6, 7 and 9 (for key, *vide supra*) showed no appreciable carbonyl absorption (less than 0·25 per cent). Number 8 (Glaxo sample 5989) had a band at 1744 cm.$^{-1}$, assigned to a 17-ketone, equivalent to about 1 per cent of such an impurity. Number 11 (Glaxo sample 1201) had three bands at 1744, 1710 and 1674 cm$^{-1}$ ; these indicate about 9 per cent of a 17-ketone, 4 per cent of a 20-ketone (or possibly in a 6-membered ring) and 3 per cent of an $\alpha\beta$ unsaturated ketone, probably a $\Delta^4$ : 3-ketone. Number 5, which was the least soluble fraction obtained by fractional crystallisation of this latter commercial sample had the same bands but reduced in intensity to about $\frac{1}{3}$ of the original, indicating a considerable purification of the cholesterol. The most soluble fraction in this crystallisation and used for the test in Section D, Table II (number 13) contained the impurities concentrated about five-fold. The progress of this purification procedure can be followed in Fig. 7, which shows the relevant spectra.

By comparison with the findings from the ultra-violet spectra, absorption at 235 m$\mu$ appears to correspond to carbonyl absorption in the infra-red, but absorption at 251m$\mu$, as in number 4, does not correspond to such absorption in the infra-red ; the main impurity in 4 must therefore be of non-carbonyl origin.

All infra-red spectra were measured in $CS_2$ solution in 1 to 3 mm. cells on a Perkin Elmer 12C spectrometer.

### REFERENCES.

JONES, R. N., HUMPHRIES, P., AND DOBRINER, K.—(1950) *J. Amer. Chem. Soc.*, **72**, 956.
*Idem*, RAMSAY, D. A., KEIR, D. S., AND DOBRINER, K.—(1952) *Ibid.*, **74**, 80.